# Effect of glutathione-stabilized silver nanoparticles on expression of *las I* and *las R* of the genes in *Pseudomonas aeruginosa* strains

**DOI:** 10.1186/s40001-020-00415-4

**Published:** 2020-05-20

**Authors:** Mina Pourmbarak Mahnaie, Hassan Mahmoudi

**Affiliations:** 1 Islamic Azad University,North Tehran Branch, Tehran, Iran; 2 Department of Microbiology, Faculty of Medicine, Hamadan University of Medical Sciences, Hamadan, Iran

**Keywords:** Silver nanoparticles, Glutathione, *Eucalyptus camaldulensis*, Green synthesis

## Abstract

**Background:**

Biofilm formation is regarded as a significant factor in the establishment of infections caused by *Pseudomonas aeruginosa*. *P. aeruginosa* is one of the most important causes of nosocomial infections. Today silver nanoparticles (Ag-NPs) are used as antimicrobials due to their well-known, chemical, biological, and physical properties. Exposure to nanoparticles could inhibit colonization of new bacteria onto the biofilm.

**Methods:**

In the present work, the green synthesis of Ag-NPs was performed using the alcoholic extract of *Eucalyptus camaldulensis*. Ag-NPs and glutathione-stabilized silver nanoparticles (GSH–Ag-NPs) were characterized using X-ray diffraction (XRD), dynamic light scattering (DLS), scanning electron microscope (SEM), and carbon, nitrogen, and hydrogen (CNH) and Fourier transform infrared spectroscopy (FTIR) techniques were applied to investigate the structure of the modified nanoparticles. Then, the antimicrobial and antibiofilm potential of the prepared Ag-NPs and GSH–Ag-NPs against *P. aeruginosa* strains was evaluated using microbroth dilution method and their effects on the expression of *las I* and *las R* genes.

**Results:**

In this study, a total of 50 *P. aeruginosa* isolates were recovered from clinical samples. According to the results, the minimum inhibitory concentration (MIC) and minimum bactericidal concentration (MBC) value of Ag-NPs against *P. aeruginosa* was determined to be 512–256 μg/ml, respectively, while the MIC and MBC value of GS–Ag-NPs against *P. aeruginosa* clinical strains was determined in a range of 128–256 μg/ml and 256–512 μg/ml, respectively. The mean expression level in *las R, las I* genes in *P. aeruginosa* strains treated with ½ MIC of Ag-NPs was decreased by −5.7 and −8fold, respectively. The mean expression levels of *las R, las I* genes in *P. aeruginosa* strains treated with ½ MIC of GS–Ag-NPs were decreased by −8.7 and −10fold, respectively (*P* < 0.05).

**Conclusions:**

The results of our study showed that Ag-NPs and GS–Ag-NPs are highly effective against *P. aeruginosa* strains. Moreover, this study also proves the promising potential of using nanoparticles as anti-biofilm formation and antibacterial agents.

## Background

*Pseudomonas aeruginosa* is one of the most important causes of nosocomial infections especially in patients with cystic fibrosis, burns. Sepsis caused by *P. aeruginosa* is a serious complication in burn infections [1, 2]. In recent years, the widespread use of antibiotics has caused the emergence of *P. aeruginosa* strains that are resistant to broad-spectrum antibiotics from different classes. So far, the presence of multidrug-resistant (MDR) *P. aeruginosa* strains is a major problem in the treatment of infections caused by MDR in important hospital wards such as the burn unit and special care unit (ICU) [3]. Biofilms estimated to be related with 65% of nosocomial infections, and are considered as the major problem in medication because antibacterial agents such as disinfectants, heat, drying are not able to eliminate bacterial biofilms. Main reasons for the antibiotic resistance of biofilms are the limitation of antibiotic entry, reduced metabolism and slow growth, resistance genes. After biofilm exposure to antibiotics, resistant cell population leads to phenotypic adaptation [4–6]. Bacterial quorum sensing (QS) affects the overall process of biofilm development. The *Lux IR*-type QS systems are in *P.* *aeruginosa*. There are several virulence factors in *P. aeruginosa* regulated by two separate *Lux* systems including *las* and *Rhl* [7, 8]*. P.* *aeruginosa* uses its two major *Las I/R* and *Rhl/I/R* QS systems, the *Las* system regulating in a cascade the *Rhl*. These systems are responsible for synthesizing self-induced molecules called AI1 and AI2, respectively. These AIs are identified and interconnected by cytoplasmic transcription factors called *Las R* and *Rhl R*, respectively [8, 9]. Thus, in the search for novel antibacterial strategies, the use of nanoparticle agents has developed as a hopeful candidate. One of the applications of nanoparticles is their use as antimicrobial agents. Silver nanoparticles (Ag-NPs) are extensively used due to their small size. Ag-NPs have received much attention because of their anti-microbial and anti-inflammatory effects. Ag-NPs offer unique physicochemical characteristics such as good conductivity, chemical stability, catalytic activity and antibacterial and anti-inflammatory effects [10, 11]. The mechanism of the antibacterial effect of these NPs is due to the attachment to the surface of the cell membrane and disruption of permeability and respiratory functions of cells and inactivating membrane proteins. These NPs can also pass through the cell wall and/or the membranes of bacteria and bind to bacterial DNA and disrupt DNA replication. Furthermore, they can interfere in ribosomal function to translate mRNA into protein forms, and so they activate cytochrome B proteins [12].

Several methods have been used for green synthesis of Ag-NPs by using biological reducing material such as plant extracts and bacteria. Among these biological agents, plant extracts are desirable due to their low cost and accessibility. In the green methods, NPs are made up of plants, algae, etc. On the other hand, for increasing the absorption capacity of the drug on the surface of the NPs, they can be covered with various materials [10, 11]. *Eucalyptus* is one of the most famous herbs in the *Myrtaceae* family, and its antimicrobial activity has gained considerable attention since ancient times. This plant is rich in polyphenols and terpenoids, and the main components of its leaf include eucalyptol or cineol (70 to 80%). The members of this family are a major source of essential oils with broad biological activities, including anti-oxidant, anti-bacterial and antifungal properties [10, 13]. Glutathione (GSH) is a tripeptide (γ-Glu-Cys-Gly) which contains a –SH group. This peptide can be a reducing agent and coating agent, and can produce uniformly water-soluble nanoparticles to easily attach antibiotics. This characteristic has an important role in medical applications [14]. Thus, in the present study, for the first time, we evaluate antibacterial and antibiofilm potential of the Ag-NPs and Ag-NPs functionalized with GSH against *P. aeruginosa* strains using the phenotypic method and associate it with the expression level of the *las R* and *las I* genes.

## Methods

### Bacterial strains and culture conditions

A total of 50 *P. aeruginosa* strains were randomly collected from clinical samples, including wounds, blood, urine and sputum of patients admitted to a teaching hospital of Tehran University of Medical Sciences during the years 2017 and 2018. The isolated *P. aeruginosa* strains were confirmed using Gram-staining; catalase and oxidative–fermentative tests, growth on a MacConkey agar at 44 °C and oxidase and urease tests; Simon citrate and Kligler’s iron agar (KIA) and sulfide indole motility test [15]. Standard *P. aeruginosa* strains named PAO1 were applied as positive control.

### Biofilm formation assays

The biofilm formation of the isolated *P. aeruginosa* strains were analyzed by using the microtiter plate assay [15]. In summary, the *P. aeruginosa* isolates were cultured in 5 ml of tryptic soy broth (TSB) medium containing 1% glucose and incubated at 37° C for 24 h. The cultures were diluted 1:100 in TSB medium. Sterile flat-bottomed 96-well polystyrene microtiter plates were inoculated with 125 µl bacterial suspension (final concentration 1 × 10^7^ colony-forming unit (CFU)/μl) and incubated for 24 h at 37 °C. After incubation, the wells were washed in triplicate with 250 μl distilled water and then dried. Afterward, 200 μl of methanol was added to the wells for fixation. Next, 200 μl of 1% crystal violet solution was added to each well for about 10–15 min. After staining, each wall was washed three times with 250 μl of distilled water, and then dried with 125 µl of 30% acetic acid in water. The absorbance of the samples was read using an enzyme-linked immunosorbent assay (ELISA) at a wavelength of 570 nm. TSB culture medium without bacteria was used as a negative control. The interpretation of the testing results of biofilm production was carried out according to the following equation [15]:

$${\text{ODc}}{\mkern 1mu} = {\mkern 1mu} {\text{average OD of negative control}}{\mkern 1mu} + \left( {{\mkern 1mu} 3{\mkern 1mu} \times {\mkern 1mu} {\text{SD of negative control}}} \right),$$

where ODc is the optical density cut-off value.

### Detection of *las R* and *las I* genes

DNA extraction from the *P. aeruginosa isolates* were performed using the Favorgen Biotech Corp (Taiwan) according to the protocol provided by the manufacturer. DNA was quantified by spectrophotometry (Thermo Scientific™ Nano Drop 2000c) over the wavelength range 260–280 nm. Polymerase chain reaction (PCR) was performed to identification the *lasR* and *lasI* genes and a reference gene (*16s rRNA*). The primers are presented in Table 1. The parameters for the amplification were 35 cycles of denaturation at 94 °C for 1 min, annealing at 60 °C for 1 min, and extension at 72 °C for 2 min. The *P. aeruginosa* strain PA01 was used as the positive control.

**Table 1:** Primers used in this study

Target gene	Sequence primer (5′→3′)		Amplicon size (bp)	Annealing temperature	References
*las R*	**F**	AAGTGGAAAATTGGAGTGGAG	130	60 °C	[16]
	**R**	GTAGTTGCCGACGACGATGAAG			
*las I*	**F**	CGTGCTCAAGTGTTCAAGG	294	60 °C	[17]
	**R**	TACAGTCGGAAAAGCCCAG			
*16* *s rRNA*	**F**	GGGGGATCTTCGGACCTCA	956	57 °C	[18]
	**R**	TCCTTAGAGTGCCCACCCG			

### Extract preparation

The leaves of eucalyptus plant were obtained from the Iranian Center for Genetic Reserves, which belongs to *Eucalyptus camaldulensis* species. In this study, alcoholic extract of Eucalyptus plant was used to prepare silver nanoparticles using green synthesis method. To prepare the alcoholic extract of the plant, the leaves of the eucalyptus plant were washed and dried. The dried leaves were then chopped and crashed into soft powder. Five grams of the eucalyptus was weighed, and diluted with 96% alcohol. Then, the mixture was placed on magnetic stirrer for 24 h. After this time, the extract was completely filtered with Whatman filter paper. Next, 100 ml of silver nitrate (5 M) was added to 140 ml of the extract. Then, the supernatant was removed, and the precipitated materials were stored at room temperature.

### Chemical modification of surface structure of Ag-NPs

In order to modify the structure of the Ag-NPs with glutathione amino acids, 20 cc of L-glutathione (Sigma-Aldrich) solution was diluted with 0.01 g of Ag-NP. The obtained mixture was sonicated/vortexed for 30 min, and then placed in a dark place for 5 h. The flask was placed on a rotary shaker at 640 rpm. Finally, the mixtures were centrifuged at 4000 rpm for 5 min. The supernatant was removed and the precipitated materials were collected and stored in a dry place. Then, X-ray diffraction (XRD), dynamic light scattering (DLS), scanning electron microscope **(**SEM), and carbon, nitrogen, and hydrogen (CNH) and Fourier transform infrared spectroscopy (FTIR) techniques were applied to investigate the structure of the modified nanoparticles.

### Determination of MIC and MBC of GS–Ag-NPs and Ag-NPs

MIC and MBC tests with GS–Ag-NPs and Ag-NPs were performed, using a standard broth microdilution method. In this step, 96-well microtiter plates were used for NPs. Aliquots of 100 μl of the Muller Hinton broth (MHB) was added into the wells of plate. Subsequently, in one of the plates, 100 μl of the serially diluted NPs were added in the first well. The bacterial suspension was then added to them 100 μl and the plates were incubated at 37 °C for 24 h.

### Total RNA extraction, complementary DNA (cDNA) synthesis, and real-time PCR processing

Twelve *P*. *aeruginosa* strains with the strong biofilm formation ability as well as *P*. *aeruginosa* PAO1 were used for positive control molecular investigation. *P*. *aeruginosa* strains were grown in TSB at static condition in the presence of ½ MIC of GS–Ag-NPs and Ag-NPs. After incubation for overnight at 37 °C, bacterial cells were harvested using vortexing and then, centrifugation at 5000*g* for 10 min. Bacterial cells were subjected to RNA extraction using TRIzol™ RNA extraction kit (Thermo Fisher Scientific, USA) according to the manufacturer’s instructions. Then, synthesis of cDNA was carried out using according Vivantis™ cDNA synthesis kit (Malaysia).Expression of *las I* and *las R* genes were quantified using quantitative real-time PCR (qRT-PCR) assay. The qRT-PCR assay was performed in a 20-μl reaction mixture containing 10 μl Real QPlus 2 × Master Mix Green, High ROX (Ampliqon Co, Denmark), 2 μl cDNA, 0.5 μl of (10 pmol) each forward and reverse primers (Table 1), and 6 μl of DEPC-treated water, under the thermal cycling conditions: initial denaturation at 94 °C for 5 min, followed by 40 cycles of denaturation at 94 °C for 5 s, annealing at 60 °C for 20 s, and extension at 72 °C for 30 s. Then, a final extension at 72 °C for 5 min was conducted. Negative control (contained the reagents of the reaction, but lacked cDNA), and DNA sample were included in each run as a positive control. The expression levels of *las I* and *las R* genes were calculated relative to the calibration sample an internal control *16S rRNA* to normalize the sample input. The changes in the expression level of target gene were analyzed by using the method adopted by Livak and Schmittgen [19].

### Statistical analysis

Statistical analyses were performed using SPSS v21 (IBM Corp. New York, USA). The assays were performed in triplicates. The difference between the expression of target genes in the control and treated samples was calculated by *T* test. The data were reported as duplicate and melt curve with considering a significance level of 0.05.

## Results

### Biofilm production

In the quantitative biofilm determination using the microtiter plate assay revealed that 49 isolates (98%) produced biofilm and the remaining 1 isolate did not, being distributed in the following categories: 48% (24/50) strong biofilm producer, 32% (16/50) moderate biofilm producer, and 18% (9/50) weak biofilm producer. All strains selected were based on strong-to-medium biofilm formation. Optical density values [17] of biofilm formation inhibition of the 12 isolates in the presence of ½ MIC concentration of Ag-NPs and GS–Ag-NPs are presented in Table 2.

**Table 2:** The profiles of biofilm formation in *P. aeruginosa* in ½ MIC concentration of GS–Ag-NPs and Ag-NPs (*P* < 0.05)

Isolates	Optical density [17] _570nm_		Optical density [17] _570nm_	
	Treatment	Control group	Treatment (Ag-NPs)	Control group
	(GSH–Ag-NPs) Mean ± SD		Mean ± SD	
P.S*-1	1.12 ± 0.02	3.05 ± 0.084	1.34 ± 0.06	3.05 ± 0.084
P.S -2	1.25 ± 0.05	3.75 ± 0.108	1.45 ± 0.08	3.75 ± 0.108
P. S-3	1.01 ± 0.03	2.89 ± 0.049	1.14 ± 0.04	2.89 ± 0.049
P.S-4	1.43 ± 0.1	3 ± 0.163	1.89 ± 0.12	3 ± 0.163
P.S-5	1.30 ± 0.08	2.90 ± 0.089	1.5 ± 0.08	2.90 ± 0.089
PS-6	0.90 ± 0.09	3 ± 0.143	1.02 ± 0.05	3 ± 0.143
P.S-7	0.63 ± 0.03	3.5 ± 0.301	0.73 ± 0.03	3.5 ± 0.301
P.S-8	0.55 ± 0.04	2.7 ± 0.184	0.65 ± 0.02	2.7 ± 0.184
P.S-9	0.62 ± 0.05	3.12 ± 0.273	0.75 ± 0.04	3.12 ± 0.273
P.S -10	0.76 ± 0.03	3.5 ± 0.187	0.95 ± 0.10	3.5 ± 0.187
P.S-11	0.75 ± 0.04	2.95 ± 0.075	0.84 ± 0.07	2.95 ± 0.075
P.S-12	0.63 ± 0.02	2.77 ± 0.140	0.75 ± 0.02	2.77 ± 0.140
*P. aeruginosa PAO1*	0.60 ± 0.04	3 ± 0.216	0.85 ± 0.04	3 ± 0.216

### Detection of *las I* and *las R* genes

**Fig. 1:**
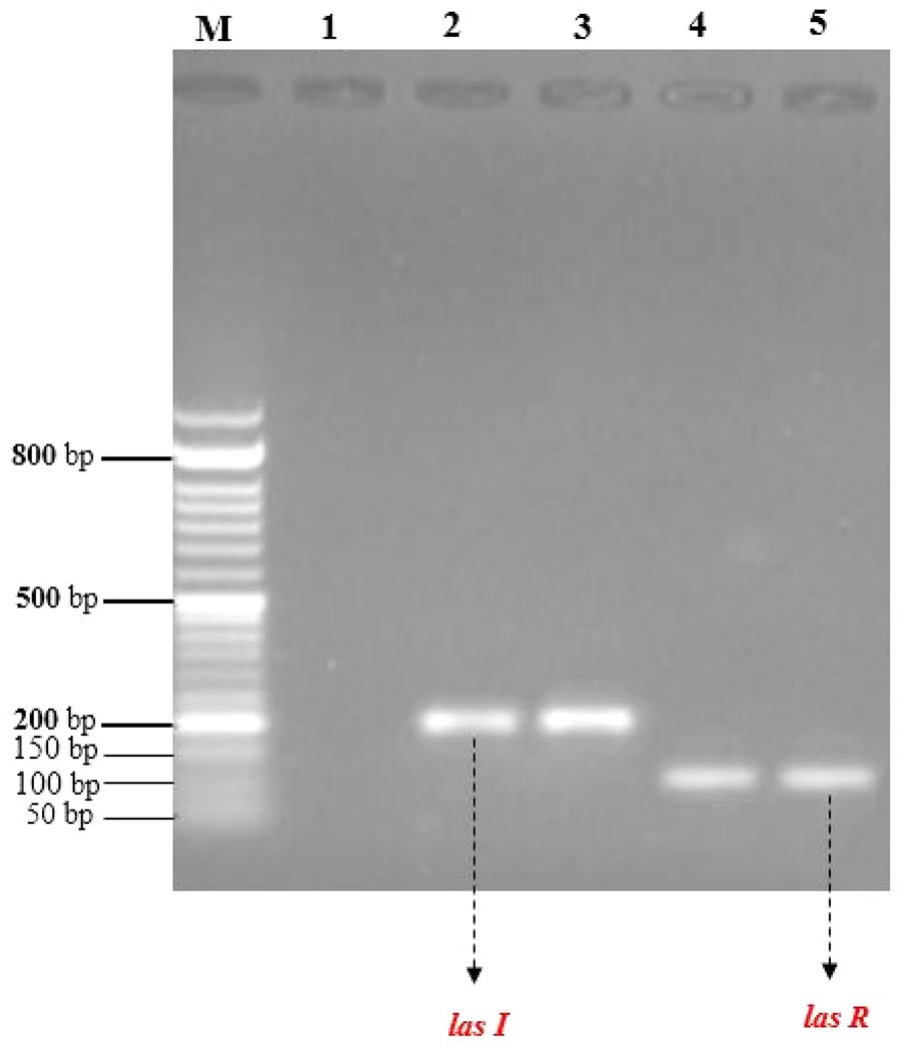
Agarose gel electrophoresis of *las I* and *las R* genes among the *P. aeruginosa* strains PCR products. M; size of DNA marker (50-bp), lane 1; (negative control) (PCR product of *qnr A* and *qnr B* gene) and lane 2; (positive control of *las I* gene), lane 3; (PCR product of *las I* gene in clinical strains), lane 4; (positive control of *las R* gene), lane 5; (PCR product of *las R* gene in clinical strains)

In this study, a high occurrence of biofilm-related genes was found, 100% and 98% of the isolates presented the *las R* and *las I* genes, respectively (Fig. 1).

### Ultraviolet–visible spectroscopy analysis

**Fig. 2:**
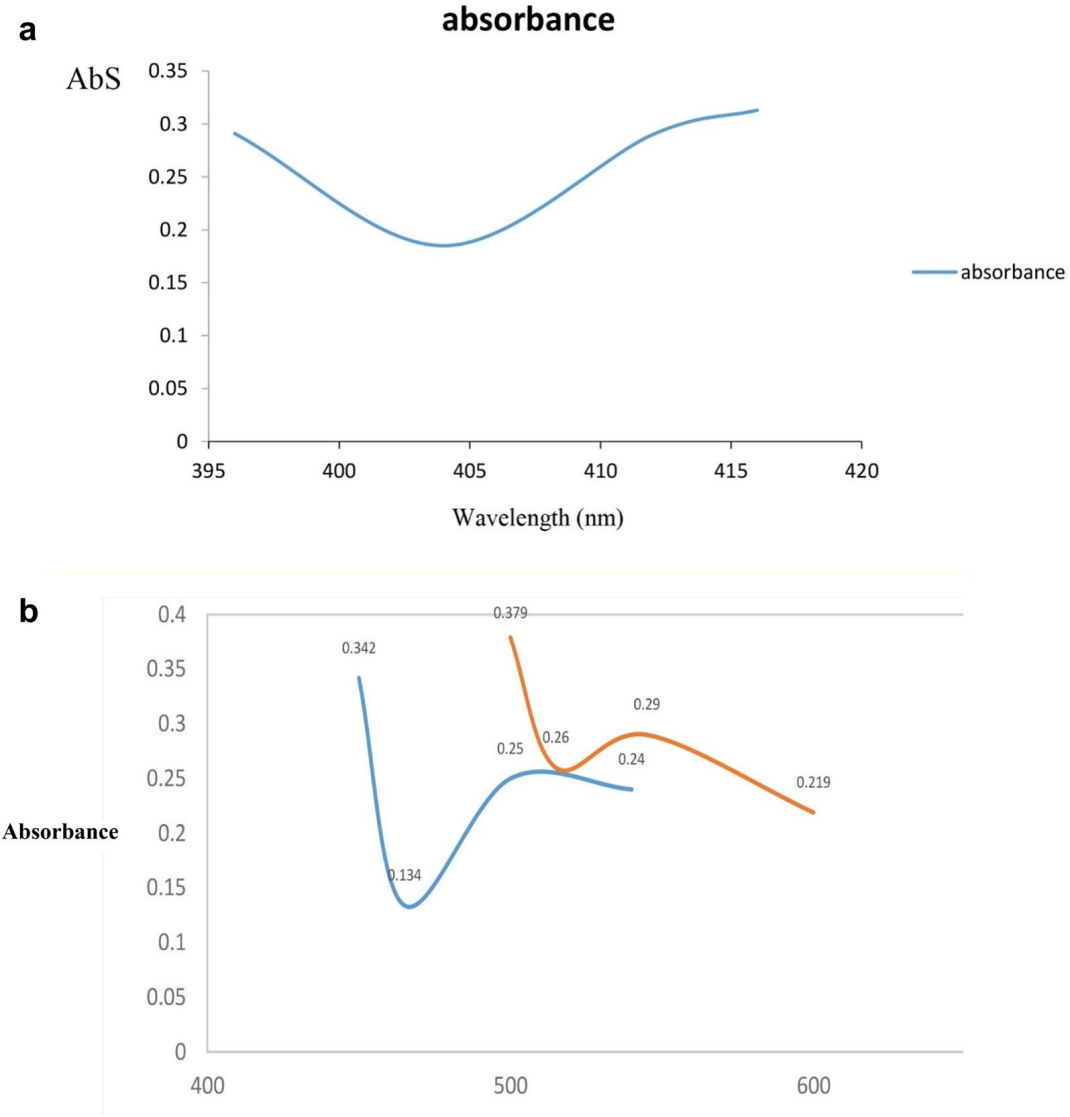
**a** Absorption spectrum of eucalyptus extract before adding silver nitrate; **b** absorption spectrum of eucalyptus extract after adding silver nitrate

An ultraviolet–visible spectroscopy was used to confirm the synthesis of Ag-NPs at wavelengths range of 350–800 nm. This analysis was performed 2 h before the addition of nitrate silver and 24 h after the addition of silver nitrate to the eucalyptus alcoholic extract. Figure 2 shows ultraviolet–visible absorption spectra of the eucalyptus alcoholic extract before and after adding silver nitrate. According to the results, the value of Lambda (**λ**) max was obtained at 450 nm.

### SEM analysis of Ag-NPs and GS–Ag-NPs

**Fig. 3:**
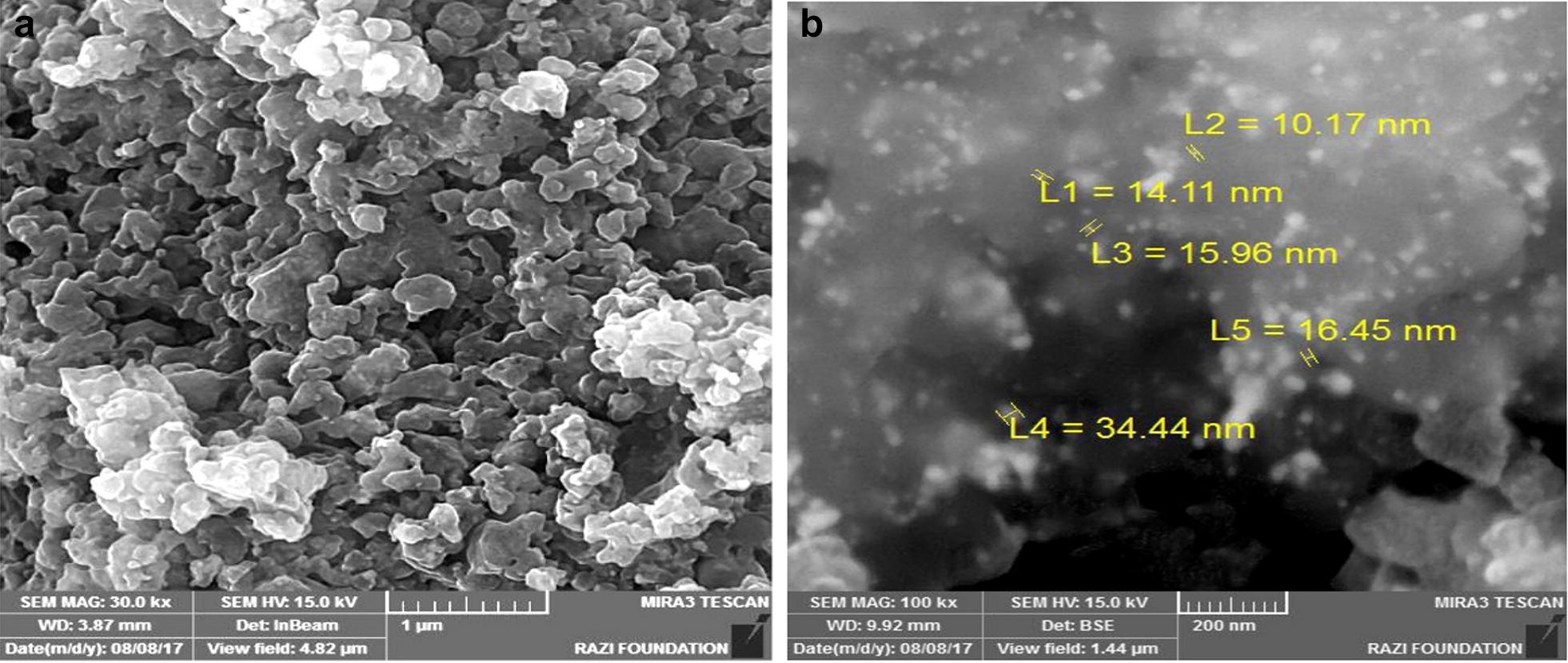
SEM analysis of **a** silver nanoparticles; **b** glutathione-stabilized silver nanoparticles (GS–Ag-NPs)

SEM image in Fig. 3 shows the morphological character of silver nanoparticles had a smooth surface. While the GS–Ag-NPs had a spherical structure. This SEM image also showed the aggregation of the silver nanoparticles.

### DLS analysis of nanoparticles

DLS is able to probe the size distribution of small particles a scale ranging from submicron down to one nanometer in solution or suspension. The results obtained in size distribution from DLS analysis show the polydispersity index (PDI) was 0.440 with a Z-average of 747.4 nm.

### XRD studies

**Fig. 4:**
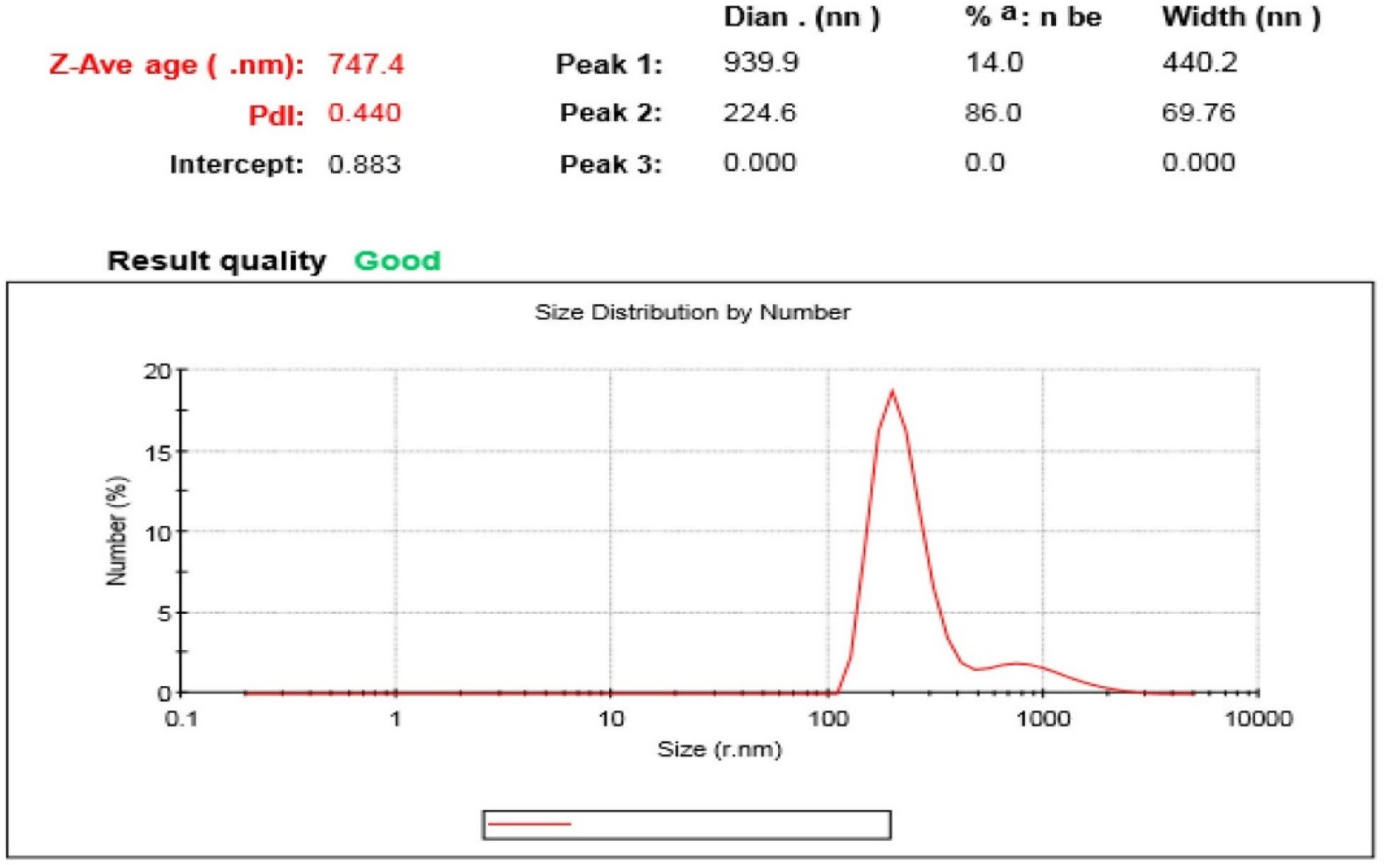
Measurement of size distribution of nanoparticles

The XRD pattern showed the main diffraction peaks of crystalline silver nanoparticles were appeared at 38.16º, 46.26º, 64.52º, 76.78º, which correspond to 111, 200, 220 and 311 planes, respectively, which are closely consistent with the standard peaks of silver nanocrystals. The XRD pattern shows that the silver nanoparticles were structurally modified with the l-glutathione amino acid. According to the results, after modification of the nanoparticles, the peak positions of the silver crystalline surface are still present and slightly shifted toward higher values, which confirmed the presence of the silver nanoparticle core modified with glutathione (Fig. 4).

#### FTIR analysis Ag-NPs, GSH and GS–Ag-NPs

**Fig. 5:**
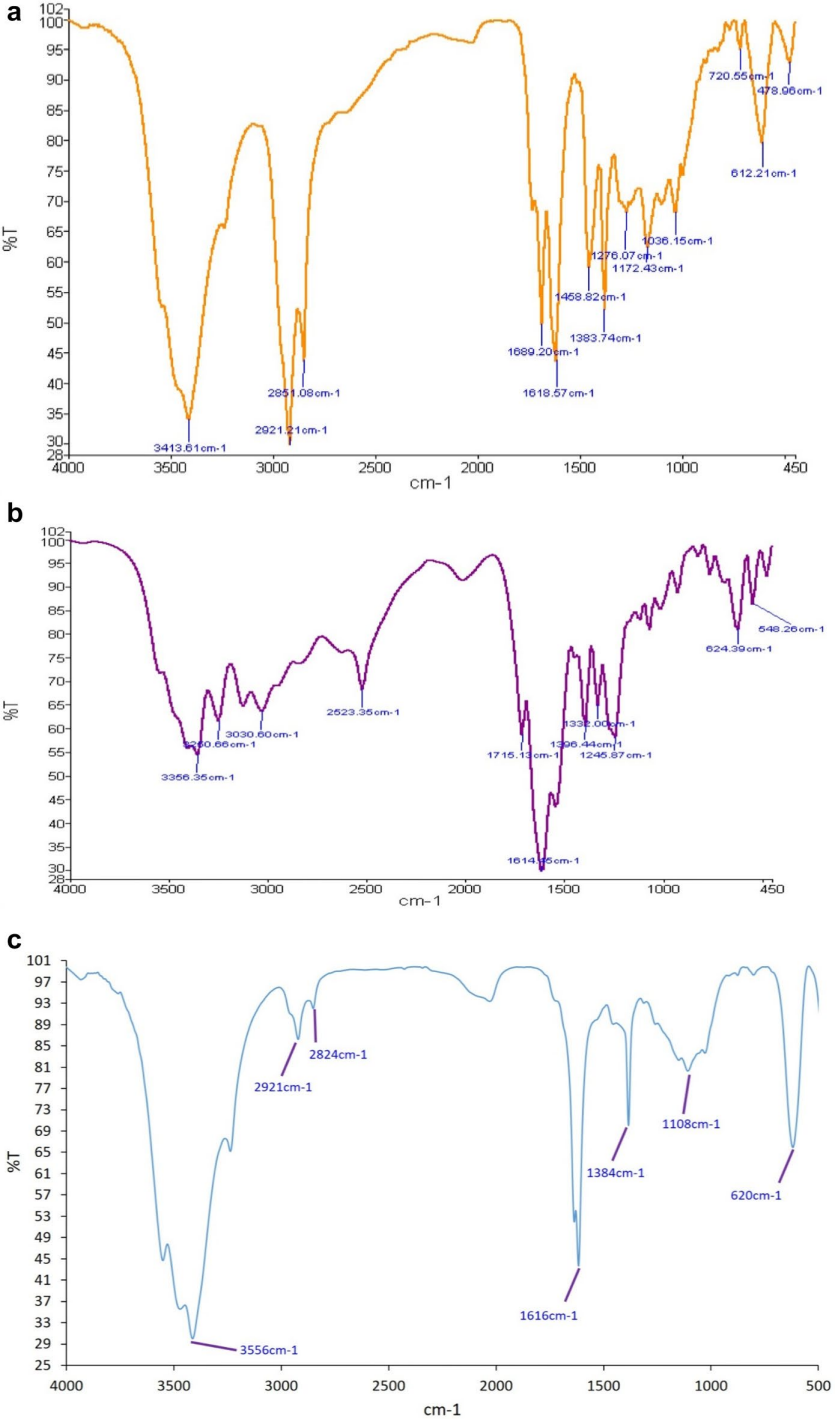
FTIR spectra of **a** Silver nanoparticles; **b** glutathione and **c** GS–Ag-NPs

The peak at 720 cm^−1^ in Fig. 5a and 624 cm^−1^ in Fig. 5b confirmed the presence of metal nanoparticles. The peaks observed at 1172, 1383, 1618 and 1458 cm^−1^ correspond to the functional groups of C–O, COO, NH, CH_2_, respectively. Also, the peak observed in 3413 cm^−1^ is related to OH, which may be attributed to plant extract residues. In the glutathione spectrum, the absorption peak at 1384 cm^−1^ is attributed to the COO functional group in the amino acid composition, and the peak at 1616 cm^−1^ is due to NH functional group and peak at 3556 cm^−1^ is due to OH-group. The adsorption peak at 2533 cm^−1^ is related to the SH group in the glutathione tripeptide (γ-Glu-Cys-Gly). In the spectrum of the modified nanoparticles, the disappearance of this peak indicated the involvement of various functional groups in bonding of the nanoparticles (Fig. 5c).

#### CNH analysis of Ag-NPs and GS–Ag-NPs

According to the CNH analysis, the percentages of carbon, hydrogen and nitrogen were 56.55%, 7.48%, and 17.1%, respectively. The carbon content of this group was related to nanoparticles. Based on the results, the carbon content of the silver-modified nanoparticles modified with L-glutathione amino acid was greater than 27.37%, hydrogen content was 88.11% and nitrogen content was 0%. The presence of carbon and hydrogen in the sample confirmed the binding of glutathione tripeptide to the surface of the silver nanoparticles, because these nanoparticles do not have such groups.

### MIC and MBC of Ag-NPs and GS–Ag-NPs against *P. aeruginosa* strains

According to the results, The MIC and MBC value of Ag-NPs against *P. aeruginosa* was determined 256 and 512 μg/ml, respectively. While the MIC and MBC value of GS–Ag-NPs against *P. aeruginosa* clinical strains was determined in a range of 128 and 256 μg/m, respectively (Table 3).

**Table 3:** Determination of MIC and MBC of Ag-NPs and GS–Ag-NPs agents against *P. aeruginosa* strains

Bacterial strains	MIC (µg/ml)		MBC (µg/ml)	
	Ag-NPs	GS–Ag-NPs	Ag-NPs	GS–Ag-NPs
*P. aeruginosa* PAO1	128	128	256	256
P.S*1	256	128	512	256
P.S2	256	128	512	256
P.S3	256	256	512	512
P.S4	256	128	512	256
P.S5	256	128	512	256
P.S6	256	256	512	512
P.S7	256	128	512	256
P.S8	256	128	512	256
P.S9	256	128	512	256
P.S10	256	256	512	512
P.S11	256	128	512	256
P.S12	256	256	512	512

*PS: *P. aeruginosa strain*

### Effect of ½ MIC of GS–Ag-NPs on the expression of genes associated with biofilm production (las I, las R)

After treatment of the isolates with ½ MIC of Ag-NPs modified with l-glutathione, the RNA was first extracted, and then the cDNA was synthesized. Next, the quantitative expression of the gene-associated biofilm *(Las I, Las R)* and the *16S rRNA* reference gene were measured using the real-time PCR. After treatment of *P. aeruginosa* strains with ½ MIC of the modified silver nanoparticles, they were treated with L-glutathione as well. A significant decrease was observed in the gene expression of *Las R* and *Las I* compared with the untreated groups (*P* < 0.05). The mean expression level in *Las R, Las I* genes in *P. aeruginosa* strains treated with ½ MIC of Ag-NPs were decreased by 5.7 and eightfold, respectively (*P* < 0.05).The mean expression level in *Las R, Las I* genes in *P. aeruginosa* strains treated with ½ MIC of GS–Ag-NPs were decreased by 8.7- and 10-fold, respectively (*P* < 0.05).

## Discussion

Bacterial biofilm formation in healthcare is a significant problem in medicine. Biofilm formation by bacteria causes higher resistance to antimicrobials due to the decrease of antimicrobial penetration and easier exchange of resistance genes between bacteria. Therefore, biofilm formation is regarded as an essential pathogenicity determinant and the resulting infections are challenging to treat [20]. The world spread of antibiotic resistance has increased the need to develop new antimicrobial agents. Ag-NPs have gained much attention as a suitable option for eradicating biofilms and antimicrobial agents [21]. The key cause of antibacterial behavior of such NPs could be attributed for the charge transfer mechanisms taking place between bacteria and the NPs. For the reason that of these characteristics such as, coating/capping, particle composition, dissolution rate, efficiency of ion release, size distribution, particle reactivity in solution, size, shape, particle morphology and agglomeration, Ag-NPs have been used widely in the health care industry, and in food storage, biomedical, and environmental applications. We accomplished a first antimicrobial assessment of synthesized Ag-NPs and glutathione-stabilized silver nanoparticles (GSH–Ag-NPs) by determination of MIC and MBC against *P. aeruginosa* strains. The obtained results prove a significant antibacterial activity of Ag-NPs and GS–Ag-NPs with bactericidal effects at the concentration of means 1024 and 256 µg/ml against *P. aeruginosa* strains, respectively (Table 2). This demonstrates the findings obtained by other researchers, which showed Ag is a potent antibiotic against a wide range of bacteria at a very low concentration without having any damaging effect on body tissues [22, 23]. Sondi et al. showed microorganisms treated with Ag-NPs exhibited the accumulation of Ag-NPs in the cell wall and the formation of “pits” in the bacterial cell walls, ultimately leading to cell death [24]. Kim et al. investigated the efficiency of the antimicrobial effects of Ag-NPs against yeast, *Staphylococcus aureus* and *E. coli*. The results show that at low concentrations of Ag-NPs, the inhibition of growth was observed in yeast, *E. coli* and *S. aureus* [25]. Silvan et al. evaluated the antimicrobial effectiveness of GSH–Ag-NPs against multidrug resistant (MDR) *Campylobacter* strains. The results obtained showed that GSH–Ag-NPs were highly effective against *Campylobacter* strains tested; the findings of this study confirm our results [26]. Thus, a covering of glutathione (GSH) increases the solubility and the capability of AgNPs to interact with the environment [23]. Also, the antimicrobial effect of Ag-NPs is due to release of Ag^+^ ions from Ag-NPs, which make an additional involvement to the bactericidal effect. Indeed, Ag-NPs where Ag^+^ is present in the Ag^0^ form also contain small concentrations of Ag+, and both Ag+ and Ag^0^ contribute to the antibacterial activity [26]. Researchers claim the antibacterial activity of the NPs may be due to the active permeability of bacterial cells through the cell wall layers or its charges. The antibacterial activity studies have demonstrated that NPs may enter the cell and attachment to cell receptors causing intracellular disintegration leading to cell death and inhibition of essential metabolic enzymes resulting in disruption of bacterial cell reproduction and respiration. Ag-NPs antibacterial mechanism works by inhibiting O_2_ metabolism, which finally kills the microbes in a very short time [27]. Moreover, it has been reported that Ag-NPs could efficiently reduction bacterial biofilm biomass [20]. These findings indicate that the antibiofilm activity of 512 μg/ml of Ag-NPs and 256 μg/ml GSH–Ag-NPs could slightly degradation bacterial biofilm (Table 3). These findings are very similar to Mohanty et al.’s report that tested anti-biofilm activities of varying concentrations of Ag-NPs against *P. aeruginosa* that reported a reduction in biofilm formation by *P. aeruginosa* [28]. The antibiofilm activity of Ag-NPs has been demonstrated in a number of studies such as Sondi et al., Montazeri et al., Besinis et al., Gurunathan et al., Kalishwaralal et al., have demonstrated the potential anti-biofilm activity Ag-NPs against Gram-negative and Gram-positive bacterial [20, 29–31]. Franci et al. demonstrated that Ag-NPs exhibit effective biofilm inhibition of against *Pseudomonas putida* biofilms [32]. Ramasamy et al. exhibited that antibiofilm Ag-NPs decrease the biofilm formation by *P. aeruginosa* [33], Shafreen et al. reported for Ag-NPs an MIC of 300 ng/ml against a biofilm formed by *E. coli* [34].

Here, Ag-NPs not only inhibited the growth, but also the capability of the bacterium to synthesize the exopolysaccharide. This shows that Ag-NPs have the ability to block the exopolysaccharide synthesis of the bacterium then the biofilm. This inhibitory and degradation effect of Ag-NPs on the biofilm formation may due to be existence of water channels (pores) throughout the biofilm. The water channels are present for nutrient transportation; Ag-NPs may directly diffuse through the exopolysaccharide layer through the pores and may impart antibacterial actions [35].

In this study, we showed that exposure of *P. aeruginosa* strains to ½ MIC concentration of GSH–Ag-NPs significantly reduced expression of both *las I* and *las R* genes reduced compared to the control sample, which could be regarded as the main cause of biofilm inhibition between *P. aeruginosa* strains (*P* < 0.05). Our results showed that GSH–Ag-NPs not only reduced biofilm formation ability of *P. aeruginosa* strains, but also reduced the expression of the main genes associated with biofilm formation. Hentzer et al. wherein HFs have shown antiquorum-sensing activity in *P. aeruginosa* by inhibiting the expression of *fabH2* gene, our results are consistent with this the report [36]. Nejabatdoust et al. showed that functionalization of ZnO NPs with Tsc could significantly increase efficiently reduced expression of the major efflux pump genes in MDR*S. aureus* strains [37]. Consequently, penetration of Ag-NPs into the bacterial cells could interrupt several cellular functions including gene expression. In other words, the NPs inhibit cytoplasmic proteins via direct attachment which describe the reduced expression of biofilm-associated genes. Montazeri et al. reported that at sub-inhibitory concentration of Ag-NPs conjugated to thiosemicarbazide reduced expression of *ica A* and *ica D* genes the biofilm formation related between methicillin resistance *S. aureus*, the results from this study support the finding [20]. In another study, Gheidar et al., showed that exposure to Ag-NPs reduced the expression of *fnbA* and *fnbB* genes [38]. In addition to the all the mentioned mechanisms of biofilm inhibition in *P. aeruginosa*, internalization of the NPs into the bacterial cells and inhibition of cellular components may affect expression of a different set of biofilm association genes, which need further research.

## Conclusion

Ag-NPs can be utilized for beneficial biological application. Glutathione functionalization of noble metal Ag-NPs may improve their biological activity. The Ag-NPs thus prepared tend to aggregate together upon addition of Ag^+^ due to the strong coordination bond between Ag^+^ and –NH2, –COOH of glutathione modifier. The findings of this study indicated that the glutathione-modified silver nanoparticles at a concentration of ½ MIC reduced the expression of *las I* and *las R* genes associated with biofilm production in *P. aeruginosa.* Nevertheless, further studies must be conducted to assess the toxicity of the NPs in in vitro and in vivo conditions.

## Data Availability

Not applicable.

## Abbreviations

Ag-NPs: Silver nanoparticles
XRD: X-ray diffraction
DLS: Dynamic light scattering
SEM: Scanning electron microscope
CNH: Carbon, nitrogen, and hydrogen
FTIR: Fourier transform infrared spectroscopy
GSH–Ag-NPs: Glutathione-stabilized silver nanoparticles
MDR: Multidrug resistant
ICU: Special care unit
QS: Quorum sensing
GSH: Glutathione
OF: Oxidative-fermentative
KIA: Kligler’s iron agar
SIM: Sulfide indole motility
TSB: Tryptic soy broth
CFU: Colony forming count
ELISA: Enzyme-linked immunosorbent assay
ODc: Optical density cut-off
PCR: Polymerase chain reaction
MIC: Minimum inhibitory concentration
MHB: Muller Hinton broth
cDNA: Complementary DNA
ROS: Reactive oxygen species
